# Protective Effect of Lemon Peel Polyphenols on Oxidative Stress-Induced Damage to Human Keratinocyte HaCaT Cells Through Activation of the Nrf2/HO-1 Signaling Pathway

**DOI:** 10.3389/fnut.2020.606776

**Published:** 2021-01-18

**Authors:** Xi Gao, Diru Xu, Xinyue Zhang, Hengguang Zhao

**Affiliations:** Department of Dermato-Venereology, University-Town Hospital of Chongqing Medical University, Chongqing, China

**Keywords:** lemon peel, polyphenol, HaCaT cells, mRNA, HPLC

## Abstract

Lemon peel can be used as traditional Chinese medicine. Flavonoids are the most important components in lemon peel, which can be developed as natural medicine without side effects. This study investigated the protective effect of lemon peel polyphenols (LPP) on human keratinocyte HaCaT cells under oxidative stress. The active components of LPP were determined by high performance liquid chromatography. The abilities of LPP to scavenge DPPH and ABTS+ free radicals were studied for detection of antioxidation *in vitro*. Cell survival rates were determined by MTT assay. The antioxidant enzyme activity and antioxidant index of cells were determined using kit. The mRNA and protein expression of cells were determined by qPCR and western blot. The ability of LPP to scavenge DPPH and ABTS^+^ free radicals were stronger than those of vitamin C (Vc) at the same concentration. As expected, compared with the normal group of cells, the model group had decreased cell survival, increased lactate dehydrogenase (LDH), decreased levels of superoxide dismutase (SOD), catalase (CAT) and glutathione (GSH), and increased malondialdehyde (MDA) content. qPCR and western blot results indicated that the expression of Bcl-2-related X protein (Bax), caspases-3, erythroid-derived nuclear factor 2-related factor 2 (Nrf2), and heme oxygenase-1 (HO-1) were decreased and the expression of B-cell lymphoma-2 (Bcl-2) was increased in the model group, compared with the normal group. LPP treatment improved cell survival rate, reduced intracellular LDH and MDA levels, increased intracellular SOD, CAT, GSH levels, down-regulated Bax, caspases-3, Nrf2, HO-1 expression, and up-regulated Bcl-2 expression. Component analyses found that LPP contains gallic acid, neochlorogenic acid, (+)-catechin, caffeic acid, (−)-Catechin gallate, isochlorogenic acid A, rosmarinic acid, and protocatechuic acid. LPP was found to regulate the Nrf2/HO-1 signaling pathway through 8 active substances to protect HaCaT cells against oxidative stress *in vitro*.

## Introduction

Lemon is a high-yielding crop that is widely consumed as a fruit and processed into a variety of juice, jam, and other food products (1). At present, it is planted in all provinces in southwest China, with Sichuan as the greatest produce (2). Lemon pulp contains multiple active ingredients including dietary fiber, vitamins, flavonoids, phenolic derivatives, limonoids, minerals, and others (3). Studies have shown that lemon helps regulate blood glucose and lipids and has a therapeutic effect on cardiovascular, inflammatory, and malignant diseases such as tumors, as well as antioxidant and antiviral effects (4). Lemon pulp is commonly used, but lemon peel is not widely used in traditional processing applications because it is thick and rough. The peel is waste in food processing, and only a small amount used as a component in some traditional Chinese medicine (5). However, the lemon peel contains various active substances, including polyphenols (3), which may offer significant therapeutic benefits.

Oxidative stress due to an imbalance between oxidation and antioxidation in the body leads to oxidative damage, neutrophil inflammatory infiltration, and produces various oxidative intermediate harmful products. Free radicals produced by oxidative stress in the body are an important factor in aging and disease. The most apparent effects of skin aging are changes in color, luster, morphology, texture, and other aspects of appearance (6). Oxidative stress damages skin cells due to disruption of oxidative balance (7) and plays an important role in the occurrence of skin aging, epidermal tumors, chloasma, leucoderma, skin trauma, polymorphous light eruption, psoriasis, herpes zoster, and allergic purpura (8). A large amount of oxygen free radicals are found in skin tissues near skin lesions, which are common manifestations of skin damage. In many cases, oxidative stress results in apoptosis or necrosis of skin cells *via* multiple mechanisms (9). Nrf2 is a regulator of oxidative stress, controlling the expression of antioxidant proteins and thereby inhibiting the oxidative stress response. Similarly, the HO-1 gene is an Nrf2-dependent gene, and its products are strong antioxidants. Regulation of the Nrf2/HO-1 signaling pathway is thus an effective way to control oxidative stress (10).

In this study, the polyphenolic compounds contained in lemon peel were first identified, and then an *in vitro* model of skin cell damage and oxidative stress was established. The protective effects of LPP against cellular oxidative stress was observed. A mechanistic analysis of the protective effects of LPP against oxidative stress found that it affects Nrf2/HO-1 signaling, providing a theoretical basis for potential therapeutic uses of lemon peel.

## Materials and Methods

### Extraction of Polyphenols From Lemon Peel

Lemon peel was freeze-dried, crushed, and passed through mesh 60. Fifty grams of the lemon peel powder were mixed with 480 mL of 70% ethanol and extracted in a water bath at 60°C for 4 h. The extracts were filtered, evenly passed through a column filled with AB-8 macroporous resin, collected, and evaporated by rotary evaporation to obtain LPP.

### Content Determination of Lemon Peel Polyphenols

A certain amount of chlorogenic acid standard was weighed and added into deionized water to prepare chlorogenic acid standard solution. Then 1.0 mL chlorogenic acid standard solution with different concentrations was drawn and added into a 25 mL volumetric flask. The 3.0 mL Folin-Ciocalteu reagent was added for mixing. After 5 min of reaction, 4.5 mL of saturated Na_2_CO_3_ solution was added into the volumetric flask, and the reaction was conducted at 30°C to avoid light for 30 min. The final absorbance was determined at 747 nm and the standard curve of chlorogenic acid was plotted. Lemon peel polyphenols was diluted to 10^−4^ times in gradient. The absorbance value of lemon peel polyphenols was determined according to the above method. The polyphenol content of lemon peel polyphenols was calculated according to the standard curve.

### Preparation of Standard Solution

Standards of gallic acid, neochlorogenic acid, (+)-catechin, caffeic acid, (−)-Catechin gallate, isochlorogenic acid A, rosmarinic acid, and protocatechuic acid were placed in centrifuge tubes to prepare standard solutions by dissolving in methanol (1.0 mg/mL). Then, the standard solutions were filtered through an organic membrane (0.22 μm) and stored in a 1.5 mL brown vial until use.

### Detection of Extracts of Lemon Peel by HPLC

Components of LPP were detected using following chromatographic conditions: chromatographic column: Thermo Scientific Accucore C18 (4.6 mm × 150 mm, 2.6 μm); mobile phase A: 100% methanol, B: 0.5% acetic acid solution; flow rate: 0.5 mL/min; column temperature: 30°C; detector: UV-Vis; detection wavelength: 285 nm; injection volume: 10 μL. The content of each component in lemon peel was calculated by external standard method as follows: Mx = Cr × Ax/Ar × C, where, Mx (mg/g): contents of component; Cr (mg/mL): mass concentration of standard; Ax: measured peak area of sample; Ar: measured peak area of standard; and C (1.0 mg/mL): concentration of sample stock solution.

### Determination of Scavenging Ability of DPPH Radicals

First, 0.01 g DPPH reagent (Phygene Life Sciences Company, Fuzhou, Fujian, China) was dissolved in a 250 mL volumetric flask with anhydrous ethanol to adjust the concentration of DPPH to 0.1 mol/L. Then, various volumes of LPP extract stock solution were added into plugged test tubes, using ultrapure water to a total volume of 0.1 mL. Next, 4.00 mL of 0.1 mol/L DPPH-free radical solution was added, vortexed, and allowed to rest for 30 min in the dark. Anhydrous ethanol was used instead of the sample as the control group. Last, 200 μL of the final reaction solution was collected to measure absorbance at 517 nm with a spectrophotometer. The measured absorbance was used to calculate the scavenging ability of lemon peel extract on DPPH radicals by the following equation: DPPH clearance rate (%) = [1-(A_1_-A_2_)/A_0_] × 100%, where A_0_: absorbance of 0.1 mL anhydrous ethanol and 4.00 mL DPPH blank control; A_1_: absorbance of 0.1 mL sample solution and 4.00 mL DPPH solution after reaction; A_2_: absorbance of 0.1 mL sample solution and 4.00 mL anhydrous ethanol.

### Determination of Scavenging Ability of ABTS^+^ Radicals

ABTS^+^ (Phygene Life Sciences Company, Fuzhou, Fujian, China) free radical working solution was prepared by mixing 5 mL of ABTS^+^ solution (7 mmol/mM) and 88 μL of potassium perphosphate water solution (140 mmol/mL) in the dark for 12 h to stabilize free radical ions. Various volumes of an extract stock solution with the active ingredients from lemon peel were added into plugged test tubes and volume was increased to 0.1 mL with ultrapure water, followed by adding 4.00 mL of the prepared ABTS^+^ radical working solution. After thorough mixing, the reaction was carried out at room temperature for 10 min. An equal volume of ethanol with equal volume was used as the control. Absorbance was measured at 734 nm wavelength (11). The scavenging ability of lemon peel extract on ABTS^+^ radicals was calculated per the following equation: ABTS^+^ clearance rate (%) = [1-(A_1_-A_2_)/A_0_] × 100%, where A_0_: absorbance of 0.1 mL anhydrous ethanol and 4.00 mL ABTS^+^ blank control; A_1_: absorbance of 0.1 mL sample solution and 4.00 mL ABTS^+^ solution after reaction; A_2_: absorbance of 0.1 mL sample solution and 4.00 mL anhydrous ethanol.

### Cell Experiment

HaCaT cells (Procell Life Science & Technology Co., Ltd, Wuhan, Hubei, China) were cultured with DMEM medium containing 10% fetal bovine serum after resuscitation in 5% CO_2_ for 24 h to allow cells to adhere (12). After 24 h, the original medium was discarded. After adherence, HaCaT cells were cultured with DMEM medium (Solarbio Life Sciences, Beijing, China) containing H_2_O_2_ (adding 31% hydrogen peroxide solution to adjust the concentration, Sigma, St. Louis, MO, USA) at the final concentration of 20, 40, 60, 80, 100, and 120 μmol/L for 4 h. The cell survival rates were measured, and then the appropriate concentration of H_2_O_2_ was selected for subsequent experiments. Then the HaCaT cells in logarithmic growth phase were divided into five groups: normal group, model group, vitamin C (Vc) group, low-concentration LPP group (LPPL, 50 μg/mL) and high-concentration LPP group (LPPH, 100 μg/mL). HaCaT cells in the normal group were not treated but were cultured with new culture medium to maintain normal growth. The model group included HaCaT cells subjected to oxidative damage. After adherence, model HaCaT cells were cultured with DMEM medium (Solarbio Life Sciences, Beijing, China) containing H_2_O_2_ at a appropriate concentration for 4 h. HaCaT cells in the Vc group were treated identically with H_2_O_2_, and then the culture medium was discarded. The Vc cells were then further cultured with the medium containing 100 μg/mL Vc for 12 h. HaCaT cells in the LPPL and LPPH groups were treated with H_2_O_2_ and the cells were cultured with medium containing 50 and 100 μg/mL LPP, respectively, for 12 h.

### MTT Assay for Cell Viability

After the cells were treated by the procedures described in Section Cell Experiment, 20 μL MTT solution (5 g/L) was added into each well and incubated for 4 h. Then, the medium was discarded, and 150 μL of DMSO was added to each well. Absorbance was measured at 490 nm (Varioskan LUX multifunctional enzyme labeling instrument, Thermo Fisher Scientific, Inc., Waltham, MA, USA) after shaking and in the dark for 20 min. The cell survival rate was calculated as follows: cell survival rate (%) = (OD value in sample group/OD value in normal group) × 100% (13).

### Determination of LDH, SOD, MDA, GSH, and CAT Levels in Cells

After the cells were treated, cell supernatant and cells were collected, and then the cells were lysed in an ice-water bath with an ultrasonic cell disruptor (shocking for 3–5 s with 4 s intervals, repeat 3 times). Then, the LDH level in the cell supernatant was determined according to the kit's instructions, and the levels of LDH, SOD, MDA, GSH, and CAT (Solarbio Life Sciences, Beijing, China) were determined by a multifunctional microplate reader, per manufacturer's instructions.

### qPCR

After treatment, the cells from all 5 groups were collected and lysed in an ice-water bath with an ultrasonic cell disruptor (shocking for 3–5 s with 4 s intervals). RNA was extracted from cells using TRIzol^TM^ (Thermo Fisher Scientific, Inc.) and diluted to 1 μg/μL. The cDNA template was obtained using 1 μL of diluted RNA solution after reverse transcription. Then, 1 μL cDNA template and 10 μL SYBR Green PCR MasterMix, 1 μL of upstream and downstream primers (Thermo Fisher Scientific, Inc., Table 1), and 7 μL of sterile distilled water were mixed, reacted at 95°C for 60 cycles and 95°C for 15 s in each cycle; 55°C for 30 s; 95°C for 30 s; 55°C for 35 s (Stepone Plus qPCR instrument, Thermo Fisher Scientific, Inc.). Relative gene expression was calculated using 2^−ΔΔCt^, with GAPDH as the internal reference (14).

**Table 1:** Sequences of the primers used for the *in vitro* experiment.

**Gene Name**	**Sequence**
*Bcl-2*	Forward: 5′-ATGTGTGTGGAGAGCGTCAACC-3′
	Reverse: 5′-CAGAGACAGCCAGGAGAAATCAA-3′
*Bax*	Forward: 5′-CCCGAGAGGTCTTTTTCCGAG-3′
	Reverse: 5′-CCAGCCCATGATGGTTCTGAT-3′
*Casepase-3*	Forward: 5′-CATGGAAGCGAATCAATGGACT-3′
	Reverse: 5′-CTGTACCAGACCGAGATGTCA-3′
*Nrf2*	Forward: 5′-ATTGCCTGTAAGTCCTGGTCA-3′
	Reverse: 5′-ACTGCTCTTTGGACATCATTTCG-3′
*HO-1*	Forward: 5′-AACTTTCAGAAGGGCCAGGT-3′
	Reverse: 5′-CTGGGCTCTCCTTGTTGC-3′
*GAPDH*	Forward: 5′-CTGGGCTACACTGAGCACC-3′
	Reverse: 5′-AAGTGGTCGTTGAGGGCAATG-3′

### Western Blot

After the cells were lysed with RIPA cell lysate, supernatant was separated, and protein concentration was determined by a protein assay kit. A total of 30–50 μg protein was loaded for SDS-PAGE separation (Thermo Fisher Scientific, Inc.) followed by electroblot transfer onto a nitrocellulose (NC) membrane. The membrane was sequentially blocked, washed, and labeled with primary antibodies against SOD, CAT, GSH and GSH-Px and secondary antibodies (Thermo Fisher Scientific, Inc.). Bound antibodies were detected using the chemiluminescence method [iBright FL1000 (Thermo Fisher Scientific, Inc.)] (15).

### Statistical Analysis

All experiments were conducted in triplicate to obtain an average value. Data were analyzed by SPSS 23 statistical software. One-way ANOVA was used to compare between groups. *P* < 0.05 was considered statistically significant.

## Results

### Content of LPP

According to the experimental method, the standard curve of chlorogenic acid standard solution was drawn. The regression equation of the standard curve was y = 0.226x-0.002 (*R*^2^= 0.997), y was the concentration of chlorogenic acid, and x was the absorbance value. According to the calculation of standard curve, the content of LPP (chlorogenic acid) reached 79.8%.

### Ability of LPP Scavenging DPPH and ABTS^+^ Free Radicals

**Figure 1:**
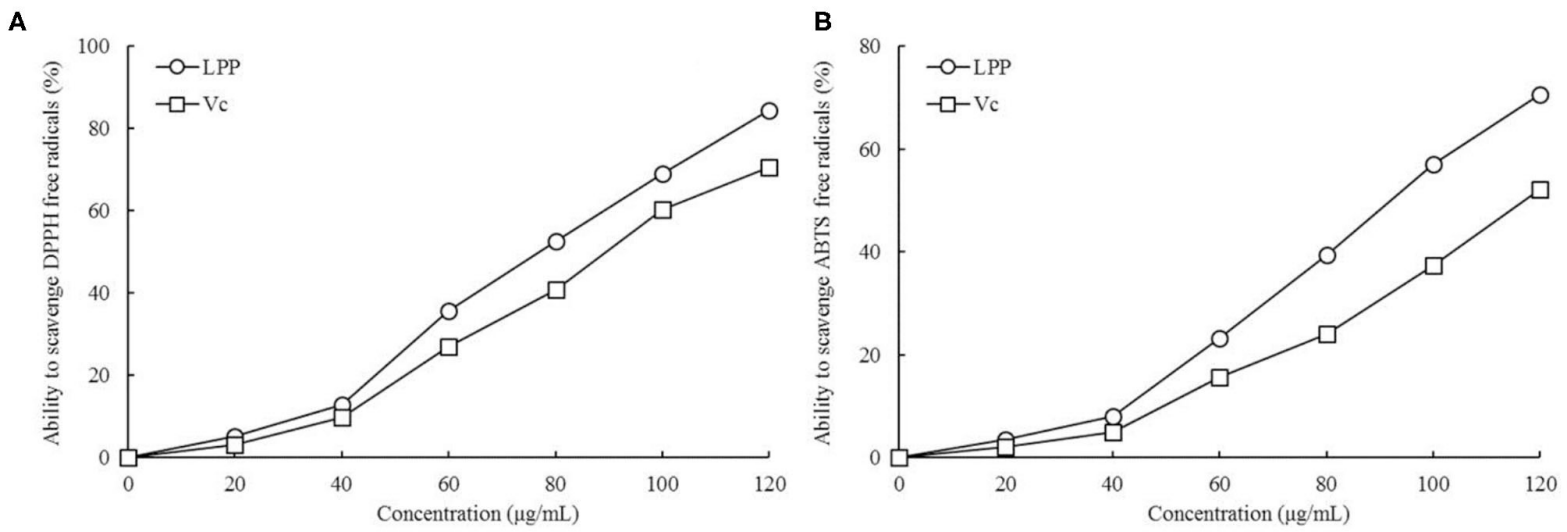
Ability to scavenge DPPH **(A)** and ABTS^+^ **(B)** free radicals of lemon peel polyphenols (LPP) and vitamin C (Vc) treatment (*n* = 6).

As illustrated in Figure 1, LPP scavenged DPPH and ABTS^+^ free radicals in a dose-dependent manner. Within the concentration range of 0-120 μg/mL, the scavenging ability increased with LPP concentration. The DPPH and ABTS^+^ free radical scavenging activities were expressed as 77.19 and 93.74 μg/mL LPP by IC_50_. The ability to scavenge DPPH and ABTS^+^ radicals in the Vc positive control group was lower than that of LPP.

### Effect of LPP on Survival Rate of HaCaT Cells With Oxidative Damage

**Figure 2:**
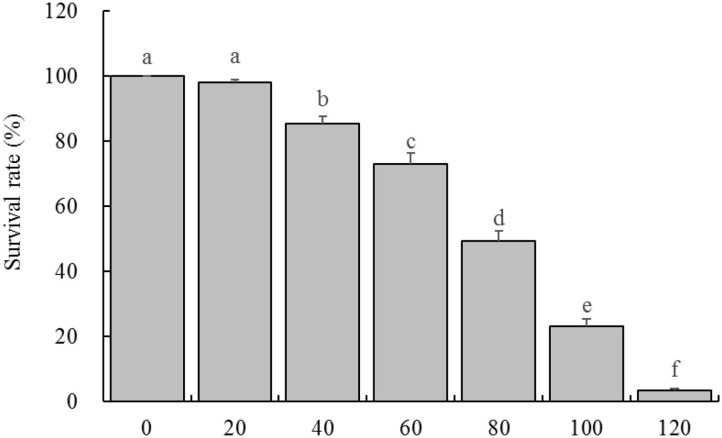
Survival rate of HaCaT cells treated with different concentrations of H_2_O_2_ (*n* = 6). “±” for standard deviation. ^a−*f*^After the Tukey's honestly significantly different test analysis, there is significant difference between the two groups with different superscript (*P* < 0.05).

By observing the effects of different concentrations of H_2_O_2_ on HaCaT cells survival rate, it was found that the concentration of 20 μg/mL had almost no effect on the cell survival rate, and the cell survival rate decreased slightly at the concentration of 40 and 60 μg/mL, while the cell survival rates were significantly decreased at the concentration of 80 and 100 μg/mL, when the concentration reached 120 μg/mL, almost all the cells died (Figure 2). Therefore, the concentration of 100 μg/mL was selected as the further experimental concentration to observe the inhibitory effect of LPP on H_2_O_2_ induced oxidative damage of cells.

**Figure 3:**
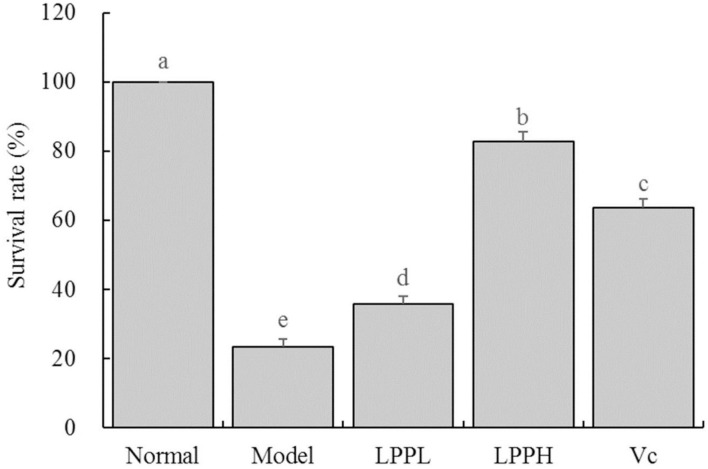
Effect of lemon peel polyphenols (LPP) on the survival rate of oxidatively damaged HaCaT cells (*n* = 6). “±” for standard deviation. ^a−*e*^After the Tukey's honestly significantly different test analysis, there is significant difference between the two groups with different superscript (*P* < 0.05).

As expected, survival rate of HaCaT cells in the model group was significantly lower than that in the normal group (Figure 3, *P* < 0.05). However, the survival rates of HaCaT cells treated with Vc (100 μg/mL) and LPP (50 and 100 μg/mL) were improved compared with the model group (*P* < 0.05). The protective effects of LPP were dose-dependent and significantly stronger than Vc at same concentration (100 μg/mL, *P* < 0.05).

### Effect of LPP on LDH Levels in Cell Supernatant

**Figure 4:**
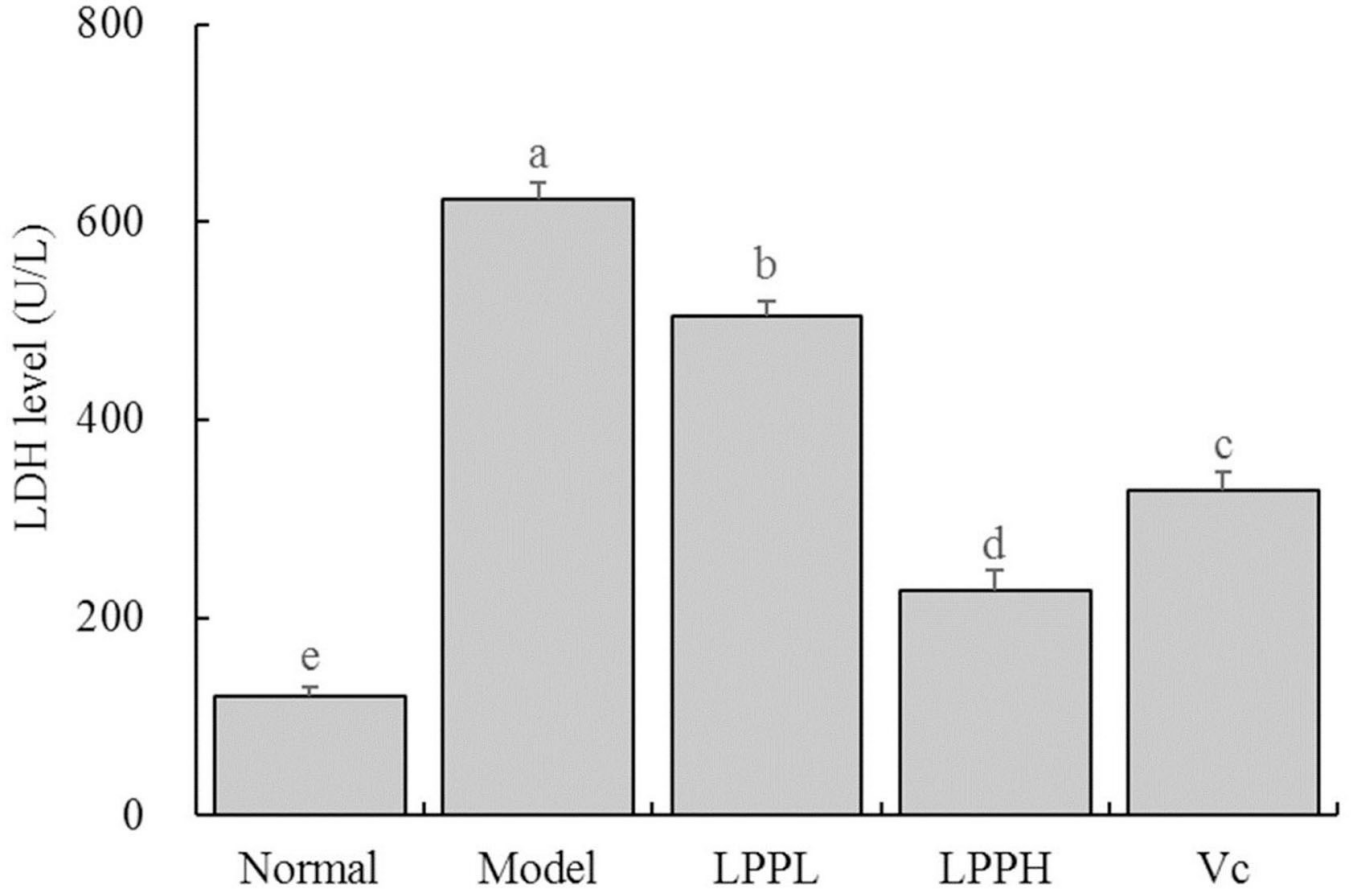
Effect of lemon peel polyphenols (LPP) on the LED level of oxidatively damaged HaCaT cells (*n* = 6). “±” for standard deviation. ^a−*e*^After the Tukey's honestly significantly different test analysis, there is significant difference between the two groups with different superscript (*P* < 0.05).

As shown in Figure 4, the level of LDH was the lowest in the normal group (120.70 ± 9.24 U/L) and highest in the model (623.55 ± 15.91 U/L). However, LPP reduced the LDH levels in cells with oxidative damage. Higher concentrations of LPP (100 μg/mL) were associated with lower LDH levels. LPP could reduce the level of LDH, regulate the LDH level of oxidative damage cells gradually return to the normal state, and the regulation effect of LPP was stronger than Vc at the same concentration of 100 μg/mL.

### Effects of LPP on SOD, MDA, GSH, and CAT Levels

Compared with the normal group, levels of SOD, GSH and CAT in HaCaT cells in the model group were significantly decreased, as expected, and the level of MDA was significantly increased (*P* < 0.05, Table 2). Compared with the model group, the levels of SOD, GSH, and CAT were increased after treatment with Vc (100 μg/mL) or LPP (50 and 100 μg/mL) treatment, and the level of MDA was decreased. This effect was also enhanced with increasing LPP concentration.

**Table 2:** SOD, CAT enzyme activities and GSH, MDA levels of oxidatively damaged HaCaT cells (*n* = 6).

**Group**	**SOD (U/gprot)**	**CAT (U/gprot)**	**GSH (μmol/mg)**	**MDA (nmol/gprot)**
Normal	208.73 ± 13.05^a^	151.31 ± 7.25^a^	71.31 ± 5.90^a^	0.47 ± 0.05^e^
Model	56.09 ± 3.63^e^	35.58 ± 4.17^e^	25.12 ± 3.59^e^	6.15 ± 0.33^a^
LPPL	97.80 ± 5.96^d^	87.00 ± 5.82^d^	35.03 ± 2.22^d^	4.99 ± 0.12^b^
LPPH	169.28 ± 9.71^b^	129.08 ± 4.38^b^	60.93 ± 2.16^B^	2.06 ± 0.12^d^
Vc	135.96 ± 10.44^c^	101.30 ± 3.55^c^	46.30 ± 3.30^C^	3.38 ± 0.13^c^

a−e *After the Tukey's honestly significantly different test analysis, there is significant difference between the two groups with different superscript (P < 0.05)*.

### Effect of LPP on Bcl-2, Bax, Caspase-3, Nrf2, and HO-1 mRNA and Protein Expression

**Figure 5:**
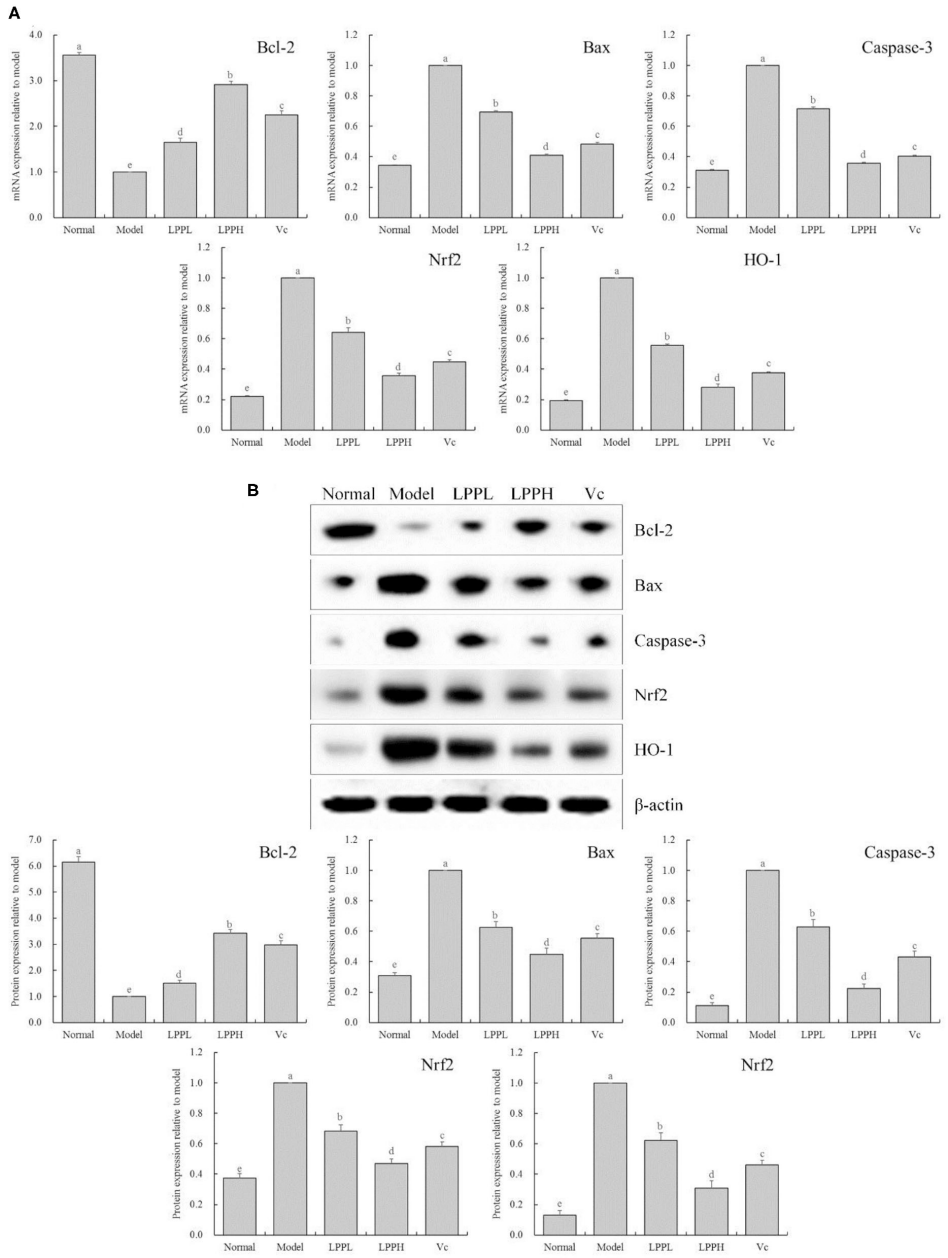
Bcl-2, Bax, Casepase-3, Nrf2 and HO-1 mRNA **(A)** and protein **(B)** expression of oxidatively damaged HaCaT cells (*n* = 3). “±” for standard deviation. ^a−*e*^After the Tukey's honestly significantly different test analysis, there is significant difference between the two groups with different superscript (*P* < 0.05).

As shown in Figure 5, compared with the normal group, the expression of Bax, Caspase-3, Nrf 2, and HO-1 in the HaCaT cells in the model group was significantly increased, and Bcl-2 was significantly decreased (*P* < 0.05). However, compared with the model group, the expression of Bcl-2 in skin cells was increased after LPP (50 and 100 μg/mL) treatment, and Bax, Caspase-3, Nrf 2, and HO-1 were decreased. These effects were strongly affected by changes in LPP concentration, and the effect of LPP (100 μg/mL) appeared to be stronger than that of Vc (100 μg/mL), in good agreement with the results from the prior experiments.

### Chemical Composition of LPP

**Figure 6:**
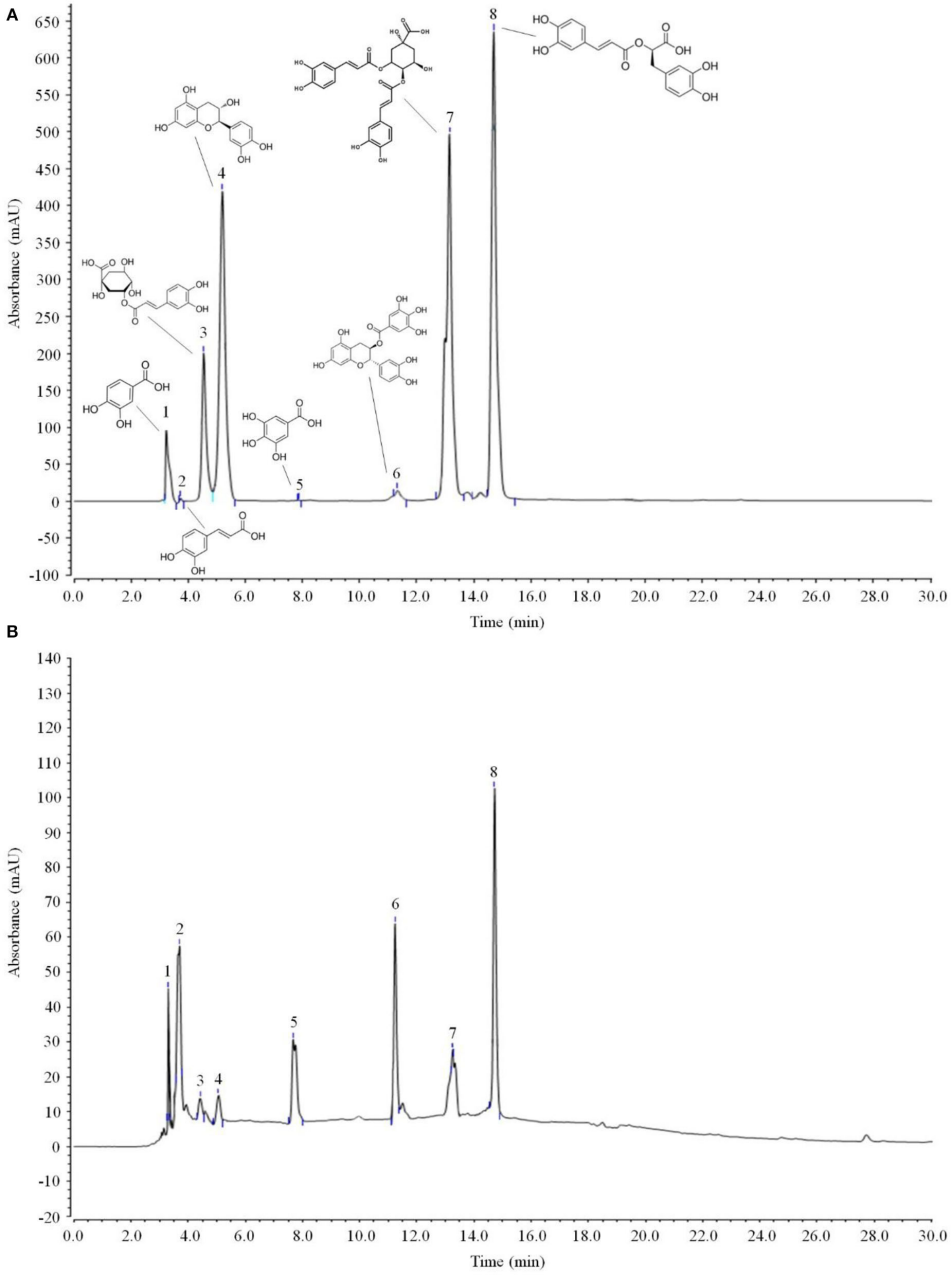
Polyphenols constituents of lemon peel (*n* = 3). **(A)** Standard chromatograms; **(B)** lemon peel polyphenols chromatograms. 1: protocatechic acid, 2: caffeic acid, 3: neochlorogenic acid, 4: (+)-catechin, 5: gallic acid, 6: (−)-catechin gallate, 7: isochlorogenic acid A, 8: rosmarinic acid.

As shown in Figure 6 and Table 3, the main active components in LPP were found to be protocatechic acid, caffeic acid, neochlorogenic acid, (+)-catechin, gallic acid, (−)-catechin gallate, isochlorogenic acid A and rosmarinic acid with contents of 20.625, 102.795, 25.575, 50.82, 17.49, 110.715, 69.96, and 255.915 mg/g, respectively.

**Table 3:** Standard curve test and polyphenols constituents of lemon peel (*n* = 3).

**Component**	**Regression equation**	**Determination coefficient (*R*^**2**^)**	**Content (mg/g)**
Protocatechic acid	y = 0.063x−0.5747	0.9883	20.625 ± 0.452
Caffeic acid	y = 0.0474x−0.4885	0.9889	102.795 ± 1.371
Neochlorogenic acid	y = 0.0312x−0.7432	0.9838	25.575 ± 0.553
(+)-Catechin	y = 0.0285x−0.2828	0.9875	50.82 ± 0.479
Gallic acid	y = 0.1034x−1.0159	0.9854	17.49 ± 0.226
(−)-Catechin gallate	y = 0.0467x−0.5053	0.9854	110.715 ± 1.105
Isochlorogenic acid A	y = 0.0244x−0.6093	0.9250	69.96 ± 1.007
Rosmarinic acid	y = 0.0386x−0.5622	0.9848	255.915 ± 1.821

## Discussion

DPPH and ABTS^+^ are both organic free radicals commonly used to quantify the antioxidant effects of active substances. In this study, LPE had significant DPPH and ABTS^+^ free radical scavenging ability *in vitro*, suggesting a strong general antioxidant effect.

As the first line of physiological defense and the largest organ of the human body, the skin plays a fundamental role in maintaining homeostasis between the body and natural environment. Changes in the body can sometimes be reflected as changes in the skin, which is closely linked to the greater health of the body. Damaged physiological function of the skin will cause skin diseases as well as potentially other diseases such as obesity, asthma, cardiovascular disease. Oxidative stress-induced damage is one of the main causes of skin damage (16). When ROS are produced in large quantities, exceeding the self-scavenging ability, it can result in damage to the lysosomes, mitochondria, and other cellular elements (17). H_2_O_2_ is one of the oxidative metabolites found *in vivo*. It reacts with free iron ions in the nucleus to produce active oxygen free radicals, resulting in cell damage that induces apoptosis and ultimately triggers cell death (18). This study confirms that H_2_O_2_ leads to a decrease in the survival rate of HaCaT cells, but LPP protects HaCaT cells and improves the survival rate after H_2_O_2_-induced oxidative damage.

Under normal physiological conditions, the LDH content in blood and body fluids is low, and intracellular LDH is released in large quantities only after damage to cell membranes (19). Thus, reducing LDH can effectively inhibit the release of LDH caused by cell damage and repair damaged cells. As the main endogenous antioxidant *in vivo*, SOD scavenges excessive oxygen free radicals, reduces mitochondrial damage, and maintains cell stability (20). GSH and CAT are also important antioxidants in the body. In the context of oxidative stress, they play a role in inhibiting oxidative damage and protecting the body (21). MDA is a product of oxidative damage, and when it is present in large quantities, it may actually enhance the degree of oxidative damage (22). We found that LPE regulates the level of LDH in culture medium as well as SOD, MDA, GSH, and CAT in cells after oxidative damage, inhibiting oxidative damage to protect HaCaT cells.

Apoptosis is a form of programmed cell death (23). The main pathway of apoptosis in skin cells is mitochondrial apoptosis, and the pro-apoptotic protein Bax and the inhibitor of apoptosis protein Bcl-2 are involved in this process, playing a role determining the degree of necrosis and apoptosis by regulating the permeability of the mitochondrial membrane (24). The Caspase family also plays an important role in apoptosis. When Bax binds to the mitochondrial membrane, the ion concentration between the internal and external mitochondrial membranes changes, leading to influx of cytochrome C into the cytoplasm, the formation of apoptotic bodies with Caspase 9, and then the activation of Caspase 3, resulting in apoptosis (25). The results of qPCR and Western blot showed that LPP increased Bcl-2, but decreased Bax and casepase3 mRNA and protein expression, suggesting that LPE protects skin cells by inhibiting the mitochondrial apoptotic pathway.

When the intracellular oxidation/antioxidant system is out of balance, excessive ROS can phosphorylate Nrf2 and dissociate it from keap-1. The activated Nrf2 translocates into the nucleus and binds with antioxidant response elements (ARE) to activate downstream antioxidant enzymes such as NAD(P)H, NQO1 and protein expression of HO-1 to maintain the balance of oxidation/oxidation and protect cells (26). As a regulator of oxidative stress, Nrf2 inhibits oxidative stress responses, through a mechanism regulated by HO-1 (27). Generally, Nrf2 and Keap1 exist in the cytoplasm as inactive dimers that can rapidly separate from Keap1 and enter the nucleus after oxidative damage.

In the nucleus, Nrf2 plays a protective role by enabling the expression of downstream antioxidant genes and the antioxidant enzyme HO-1 (28). Oxidative stress in the skin not only changes the structure and function of proteins, lipids, and DNA at the molecular level, but also activates the mitogen-activated protein kinase pathway, nuclear transcription factor pathway, signal transduction, and activating transcription factor pathways at the transcriptional level. Further, it inhibits certain signal transduction pathways, such as the Nrf2 pathway, and causes skin apoptosis and degradation of the extracellular matrix, ultimately resulting in manifestations of photodamage such as erythema, desquamation, wrinkles, and even tumors (29). Studies have shown that Nrf2 also regulates the expression of Bcl-2, SOD, and CAT and plays an anti-apoptotic and anti-oxidative role (30). In good agreement with those prior findings, we found that LPP plays an antioxidant role by increasing the mRNA and protein expression of Nrf2 and the downstream gene HO-1 *via* qPCR and Western blot, indicating that the antioxidant effects of LPP may be related to the Nrf2/HO-1 signaling pathway.

Gallic acid, neochlorogenic acid, (+)-catechin, caffeic acid, isochlorogenic acid A, rosmarinic acid, and protocatechuic acid all have strong antioxidant effects (31–37). MMP-1 is closely related to human skin aging, and its over-expression negatively affects skin health. Studies have shown that catechins can inhibit MMP-1 activity, thereby protecting the skin (38). (−)-Catechin gallate possesses a good antioxidant effect and promoting cell transport (39). Isochlorogenic acid A has a strong antioxidant and anti-inflammatory effect, inhibiting oxidative stress-induced inflammation to protect the skin (35). Damage to melanocytes in the skin may lead to insufficient production of melanin, potentially causing vitiligo. Oxidative stress is one important factor that damages skin melanocytes. Rosmarinic acid protects melanocytes and promotes the production of melanin in human epidermal melanocytes, thus maintaining skin health (40). Protocatechuic acid protects cells through both antioxidant and antimicrobial effects and inhibits the development of skin lesions (41). Gallic acid, catechin, caffeic acid, rosmarinic acid, and protocatechuic acid have thus been used as key active components of cosmetics to protect the skin. Therefore, the main protective benefits offered by LPP for the protection of skin cells against oxidation is likely derived from the activity of these compounds.

## Conclusion

In summary, we found that LPP significantly protects HaCaT cells against oxidative damage. The mechanism of this effect appears to be through regulation of the Nrf2/HO-1 signaling pathway by the 8 identified active substances, resulting in improvement of antioxidant enzymes such as SOD, GSH, and CAT in skin cells, and inhibition of apoptosis. In future work, we hope to further investigate the molecular mechanism of LPP to further clarify its targets, providing a theoretical basis for the development of LPP as a valuable therapeutic agent derived from lemon peel.

## Data Availability Statement

The original contributions presented in the study are included in the article/supplementary material, further inquiries can be directed to the corresponding author/s.

## Author Contributions

XG performed the majority of the experiments and wrote the manuscript. DX and XZ contributed to the data analysis. HZ designed, supervised the study, and checked the final manuscript.

## Conflict of Interest

The authors declare that the research was conducted in the absence of any commercial or financial relationships that could be construed as a potential conflict of interest.
